# Recent Advances in Gold Nanocluster-Based Biosensing and Therapy: A Review

**DOI:** 10.3390/molecules29071574

**Published:** 2024-04-01

**Authors:** Lu Yang, Pengqi Hou, Jingyi Wei, Bingxin Li, Aijun Gao, Zhiqin Yuan

**Affiliations:** College of Chemistry, College of Materials Science and Engineering, Beijing University of Chemical Technology, Beijing 100029, China

**Keywords:** gold nanoclusters, fluorescence, biosensing, imaging, therapy

## Abstract

Gold nanoclusters (Au NCs) with bright emission and unique chemical reactivity characters have been widely applied for optical sensing and imaging. With a combination of surface modifications, effective therapeutic treatments of tumors are realized. In this review, we summarize the recently adopted biosensing and therapy events based on Au NCs. Homogeneous and fluorometric biosensing systems toward various targets, including ions, small molecules, reactive oxygen species, biomacromolecules, cancer cells, and bacteria, in vitro and in vivo, are presented by turn-off, turn-on, and ratiometric tactics. The therapy applications are concluded in three aspects: photodynamic therapy, photothermal therapy, and as a drug carrier. The basic mechanisms and performances of these systems are introduced. Finally, this review highlights the challenges and future trend of Au NC-based biosensing and therapy systems.

## 1. Introduction

Gold, as one of the most biocompatible noble metals, has been a cornerstone in nanoscience and nanotechnology for decades. The successful preparation of gold nanocrystals marked a significant milestone in this field [1,2,3]. In order to achieve diverse goals, extensive efforts have been made to develop synthetic routes for the production of gold nanomaterials with varying sizes and shapes, such as gold nanoparticles, gold nanorods, gold nanostars, gold nanocubes, and gold nanoclusters (Au NCs), etc. [4,5,6,7,8,9,10]. Among them, Au NCs, composed of several to tens of gold atoms, exhibit discrete energy gaps and size-dependent emission properties and have been widely applied to various aspects, including detection, catalysis, energy conversion, and so on [11,12,13,14,15]. The ultrasmall size usually enables their bright fluorescence emission through d–sp transition or ligand-to-metal/metal–metal transition mechanisms [16,17]. As a consequence, Au NCs could be used for chemo/biosensing and imaging applications by recording the fluorescence signals.

Recently, biomedical diagnoses and therapy have garnered significant research interest. Toward this goal, diverse Au NCs have been explored and applied into biomedical studies [18]. Accordingly, numbers of Au NC-based sensors and therapeutic agents have been reported [19]. For example, protein-stabilized Au NCs with great biocompatibility have been widely used for bioimaging, biosensing, and therapy [20]. In addition, thiolate-capped Au NCs with a precise gold atom number also show extensive applications in both in vitro and in vivo studies [21]. We have witnessed significant advancements in the development of Au NC-based sensing probes and therapeutic agents that exhibit improved sensitivity, selectivity, and stability. It is thus crucial to provide a comprehensive summary of Au NC-based applications for junior researchers.

Despite the contribution of a few excellent review papers on Au NC-based sensors and imaging agents in the last few years [22,23,24], summarizing some of the most recent developments that focus on biosensing and therapy will benefit junior researchers to comprehend the basic rules in preparing functional Au NCs and to choose a proper modification strategy for the manufacture of advanced Au NCs. This review concentrates on the application rather than the basic introduction of Au NCs; thus, the synthesis and optical properties are not discussed since not many path-breaking approaches or markedly different properties have been reported in recently. In this review, recent developments in Au NC-based biosensing systems for various species (e.g., ions, small molecules, reactive oxygen species, biomacromolecules, cancer cells, and bacteria) are summarized. In addition, the Au NC-involved therapeutic applications are concluded in three aspects: photodynamic therapy, photothermal therapy, and as a drug carrier. To illustrate the detailed mechanisms, a few examples are presented in each described section. Finally, this review ends with the discussion of current challenges and future prospects of Au NCs for diagnosis and therapy applications.

## 2. Au NC-Involved Biosensing Applications

Fluorometric assays with a high sensitivity attract widespread concerns [25]. So far, many Au NC-based sensors have been reported for detecting various targets, such as ions, small molecules, reactive oxygen species, proteins, and DNA [26]. Meanwhile, the targeting of cancer cells and bacteria has also been realized based on Au NC probes [27]. On the basis of fluorescence signal variation trends, the sensing tactics can be classified into three categories: turn-on, turn-off, and ratiometric change. To facilitate the reading, herein, sensing targets were classified into six categories, including ions, small molecules, reactive oxygen species, biomacromolecules, cells, and bacteria [28].

### 2.1. Ions

**Figure 1:**
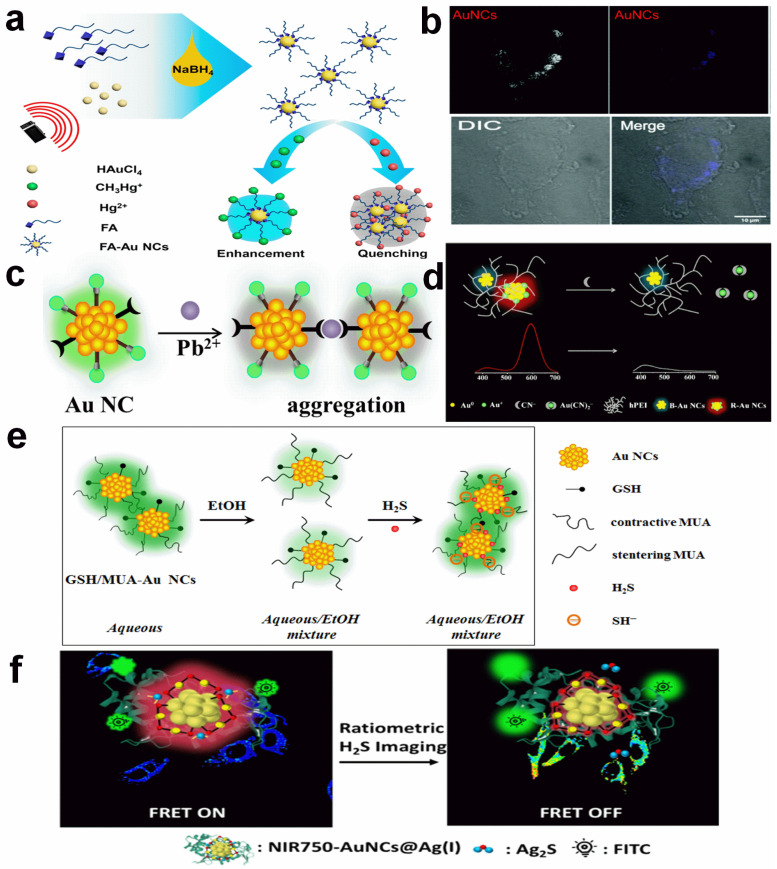
Schematic illustration of ion detection with Au NC-based probes. (**a**) Folic acid-capped Au NCs for Hg^2+^ detection. Reprinted with permission from ref. [29]. Copyright 2018, American Chemical Society. (**b**) Intracellular Hg^2+^ imaging with human serum albumin-stabilized Au NCs. Reprinted with permission from ref. [30]. Copyright 2021, Royal Society of Chemistry. (**c**) Target-induced aggregation mechanism for Pb^2+^ detection with dual-ligand-protected Au NCs. (**d**) Ratiometric CN^−^ sensing with dual-emissive Au NCs. Reprinted with permission from ref. [35]. Copyright 2020, Springer. (**e**) Turn-on sensing of H_2_S with MUA-Au NCs based on an aggregation-enhanced emission strategy. Reprinted with permission from ref. [37]. Copyright 2020, Springer. (**f**) Ratiometric H_2_S sensing with FITC-labeled lysosome-capped Au NCs. Reprinted with permission from ref. [40]. Copyright 2022, American Chemical Society.

Heavy metal ions and toxic anions have caused much research interest. In general, mercury (Hg^2+^), copper (Cu^2+^), and lead (Pb^2+^) are usually investigated [29,30,31,32,33]. For Hg^2+^, it can interact with a Au or S atom, which forms a Au-Hg alloy or Hg-S complex and destroys the structure of Au NCs. As a result, the fluorescence is quenched. For example, using folic acid-capped Au NCs (FA-Au NCs) as the probe, Yang et al. reported a rapid Hg^2+^ sensing with a LOD of 28 nM (Figure 1a) [29]. The introduction of Hg^2+^ broke the Au-S bonding and led to the aggregation of FA-Au NCs, thus resulting in fluorescence quenching. The linear response was shown in the concentration range of 100 to 1000 nM. With human serum albumin capsulation, the perception of Hg^2+^ in cancerous MDA-MB-231 cells was achieved [30]. For in vitro assays, the fluorescence of Au NCs was greatly inhibited via a d^10^-d^10^ interaction (Figure 1b). The LOD was determined to be 10 nM. Compared to Hg^2+^, Cu^2+^ always binds to the functional groups (e.g., -NH_2_, -COOH) of surface ligands, which produces photo-induced electron transfer or causes the aggression of Au NCs, subsequently quenching the fluorescence. As an example, with the insulin-capped Au NCs (Ins/Au-NCs) as the reporter, the turn-off detection of Cu^2+^ was reported by Shamsipur and co-workers [33]. The LOD and linear range toward Cu^2+^ were 7.5 nM and 0.05–1.70 μM. And the sensing of exogenous Cu^2+^ in a MCF-7 cell was also realized with Ins/Au-NCs. Through the Pb^2+^ coordination-induced aggregation, a selective and sensitive Pb^2+^ detection system was established by using dual-ligand protected Au NCs (Figure 1c) [31]. The LOD toward Pb^2+^ was calculated to be 2 nM, which is much lower than the maximum contamination level set by the U.S. Environmental Protection Agency. In addition to Hg^2+^, Cu^2+^, and Pb^2+^, Liang et al. has reported the detection of a silver ion (Ag^+^) by integrating protein encapsulation [34]. They found that the fluorescence of thiolactic acid-capped Au NCs could be enhanced by BSA adsorption, and the enhancement was further improved with the addition of Ag^+^. The LOD toward Pb^2+^ was calculated to be 40 nM.

The anionic species, including cyanide (CN^−^) and sulfide (S^2−^/HS^−^), generally show strong affinity toward the Au atom [35,36,37,38]. For instance, CN^−^ can oxidize a Au atom to a Au(CN)_2_^−^ complex with the assistance of dissolved oxygen [39]. Such a reaction has been widely used in gold mining. Through surface valence state-derived etching, Yuan et al. reported a ratiometric CN^−^ sensing platform using hyperbranched polyethyleneimine (hPEI) protected dual-emissive Au NCs (DE-Au NCs) [35]. In their work, the blue fluorescence was attributed to the d–sp transition of nearly neutral Au_8_ NCs, while the red fluorescence was assigned to the ligand-to-metal/metal–metal charge transfer of red-emissive Au NCs with a high content of surface Au(I). They disclosed that the red-emissive Au NCs with more surface Au(I) could be easily oxidized by CN^−^. In contrast, neutral Au_8_ NCs displayed a high resistance toward CN^−^ (Figure 1d). In addition, the amine-rich character of hPEI made DE-Au NCs positively charged. As a result, the DE-Au NC-based nanosensor showed satisfying selectivity toward CN^−^ over other anions. The LOD was measured to be 10 nM, and the practical detection of CN^−^ in river water and urine samples was also achieved.

Different to CN^−^, S^2−^ only binds to an Au atom and sometimes forms a Au_2_S shell on the surface of Au NCs. The surface adsorption of S^2−^ changes the charge state of Au NCs and then adjusts the dispersive status [38]. In Yuan’s report, they discovered that 11-mercaptoundecanoic acid-capped Au NCs (MUA-Au NCs) possess an unusual aggregation-enhanced emission property in an organic solvent (Figure 1e) [37]. That is, the MUA-Au NCs show high solubility in an organic solvent due the hydrophobicity of the MUA ligand with a long alkyl chain. Meanwhile, it becomes insoluble in aqueous media, forms large aggregates, and emits bright fluorescence. With this character, they constructed a turn-on S^2−^ sensing platform by using 30% EtOH solution-diluted MUA-Au NCs. The introduction of S^2−^ made MUA-Au NCs negatively charged and insoluble in 30% EtOH solution. As a consequence, large MUA-Au NC aggregates formed and the fluorescence was greatly enhanced. The highly ordered MUA layer also hindered the entrance of large molecules, eliminating the possible inference from GSH and Cys. The LOD was measured to be 35 nM, and the practical detection of S^2−^ in human serum samples was realized.

With the combination of inorganic or organic fluorophores as the references, ratiometric HS^−^ detection systems were also developed. As referred in Li’s work, with the integration of lanthanide nanoparticles and near-infrared II emitting Au NCs, ratiometric imaging of HS^−^ in the liver was achieved [36]. In addition, the liver delivery efficacy and dynamics of a H_2_S prodrug could be evaluated by a real-time imaging analysis. Through FITC labeling, Chuang et al. presented a ratiometric HS^−^ analysis method using silver ion-modified, lysosome-stabilized, near-infrared emitting Au NCs (Figure 1f) [40]. The HS^−^ approaching induced the formation of Ag_2_S, which decreases the emission of Au NCs and cuts off the energy transfer between Au NCs and FITC, resulting in the increment of a green emission of FITC. The LOD was determined to be 0.5 μM, and the practical detection of CN^−^ in river water and urine samples was also achieved. Additionally, the ratiometric imaging of exogenous and endogenous H_2_S in a HeLa cell can also be realized with such a dye-Au NC conjugate. Other anions with a strong oxidation capacity can also be detected with an Au NC probe by surface oxidation-caused fluorescence quenching [41]. Taken together, the binding affinity between ions and a gold core or surface ligand can be used to design effective Au NC-based fluorometric ion detection systems.

### 2.2. Small Molecules

In comparison to ions, small molecules are larger in size and difficult to approach at the Au NC surface because of the steric hindrance effect. For the detection of small molecules, there are three major pathways [42,43,44,45,46,47,48,49,50]. 1. Small molecules can be converted into more reactive species that interact with Au NCs and alter the fluorescence. 2. Small molecules bind to other species and interrupt the interaction between them and Au NCs, inhibiting the fluorescence variation. 3. Small molecules can react with a surface ligand, then form a complex, or produce fluorescent products, or change the charge/dispersive state, and, finally, induce fluorescence difference.

For the first pathway, substances that can be converted into hydrogen peroxide (H_2_O_2_) through enzymatic catalysis, including glucose, cholesterol, and uric acid, have caused widespread research interest [47]. In addition, dopamine (DA) is easily oxidized to orthoquinone, which quenches the fluorescence of nearby fluophores. With this mechanism, a DA analysis method was exploited using tyrosine-modified Au NCs with the assistance of a Ce^3+^-mediated assembly [43].

**Figure 2:**
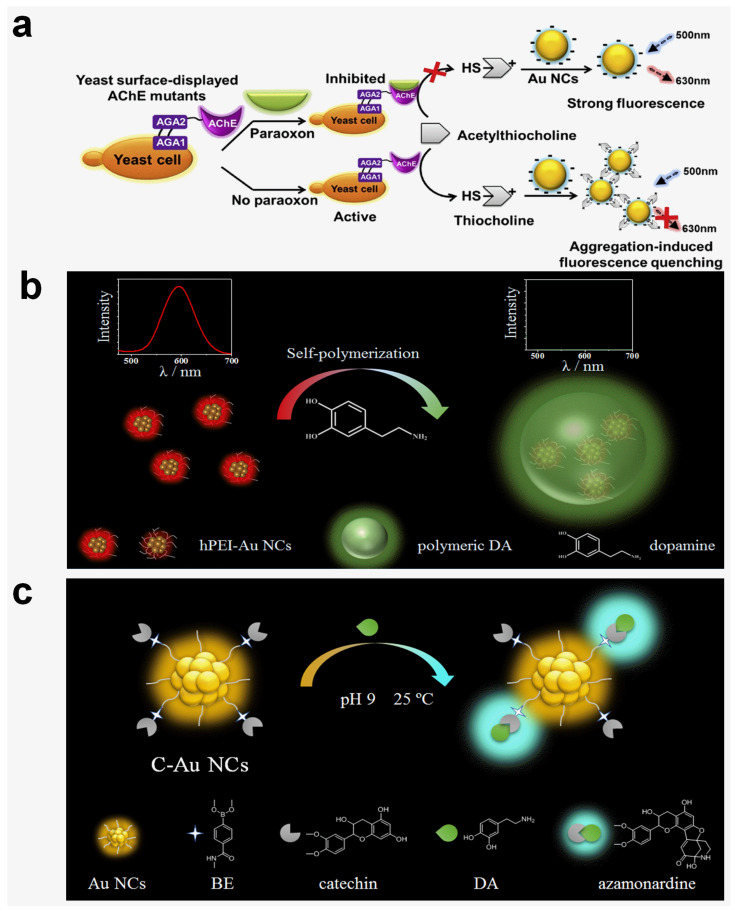
Schematic illustration of small molecule detection with Au NC-based probes. (**a**) Detection of organophosphate pesticides with protein-stabilized Au NCs by inhibiting acetylcholinesterase’s activity. Reprinted with permission from ref. [44]. Copyright 2020, Elsevier. (**b**) hPEI-Au NC-mediated ratiometric DA detection. Reprinted with permission from ref. [54]. Copyright 2022, Frontiers. (**c**) Ratiometric DA sensing with C-Au NCs by forming azamonardine products. Reprinted with permission from ref. [56]. Copyright 2022, Elsevier.

For the second pathway, enzymatic catalysis is usually involved. For example, the detection of organophosphate pesticides with protein-stabilized Au NCs was achieved by regulating the acetylcholinesterase-catalyzed hydrolysis of a thioacetylcholine ester [44]. The sensing mechanism is that the introduced organophosphate pesticides bind to acetylcholinesterase and reduce its catalytic activity, which inhibits the production of thiocholine and prevents the aggregation of Au NCs (Figure 2a). Therefore, the fluorescence intensity gradually increased with the increasing pesticide concentration. In addition to enzyme chemistry, an on–off–on strategy with a nanoquencher is also applied to develop smart Au NC-based sensors. As shown in Sang’s report, the MnO_2_ nanosheets could efficiently suppress the fluorescence of Au NCs, while the introduction of GSH destroyed the MnO_2_ nanosheets, bonded to Au NCs, and recovered the fluorescence [48]. Interestingly, despite the similar structures of cysteine (Cys) and homocysteine (Hcy), they could not cause a conspicuous fluorescence increment as GSH did. They think GSH has more electron-rich groups and a significant steric hindrance effect, and these factors benefit the fluorescence enhancement over Cys and Hcy.

Those two tactics are simple and effective, and thus, many works in response to these mechanisms have been reported to develop small molecule-sensing platforms [51]. However, the non-entrenched binding makes these systems easily affected by other environmental parameters. Thus, there is a need to explore a more powerful tool for a specific and accurate sensing application. Toward this goal, chemical reactions with high specificity are considered [52,53]. For instance, the ligand-target reaction was applied for DA sensing using an hPEI modified Au NCs (hPEI-Au NCs) [54]. The hPEI could assist the DA self-polymerization reaction, which produces polymeric DA nanoparticles with a bright green emission [55]. The Au NCs’ encapsulation weakened the proton sponge effect of the hPEI layer and promoted the DA self-polymerization reaction. In addition, the intrinsic red emission of hPEI-Au NCs was reduced because of the varied hPEI molecular conformation. Thereby, ratiometric DA sensing with high sensitivity and selectivity was achieved (Figure 2b). The LOD toward DA was measured to be 10 nM. To further boost the sensitivity, catechin-anchored Au NCs (C-Au NCs) were proposed for DA detection [56]. The ratiometric detection mechanism is based on the fact that the DA–catechin chemical reaction produces an azamonardine compound with strong fluorescence, and the intrinsic emission from C-Au NCs act as the reference (Figure 2c). The fluorescence quantum yield of the azamonardine compound is higher than 40%, thus leading to a high sensitivity. The LOD was determined to be 1.0 nM (S/N = 3) with great linearity (R^2^ = 0.995, linear range: 0 to 500 nM). As expected, the characteristic chemical reaction afforded high specificity toward DA over other possible interferents, including amino acids, metal ions, and small molecules. The DA content analysis with the C-Au NCs probe was also performed in urea and cell lysate samples with high accuracy. With the combination of linear discriminant analysis, nitrophenol analogues with similar structure and reactivity were successfully distinguished [57]. It is noticeable that the analyte–gold core interaction usually destroys the structure and quenches the fluorescence. To achieve ratiometric analysis, it is speculated that surface functionalization with a reactive ligand may contribute a high sensitivity and specificity.

### 2.3. Reactive Oxygen Species

**Figure 3:**
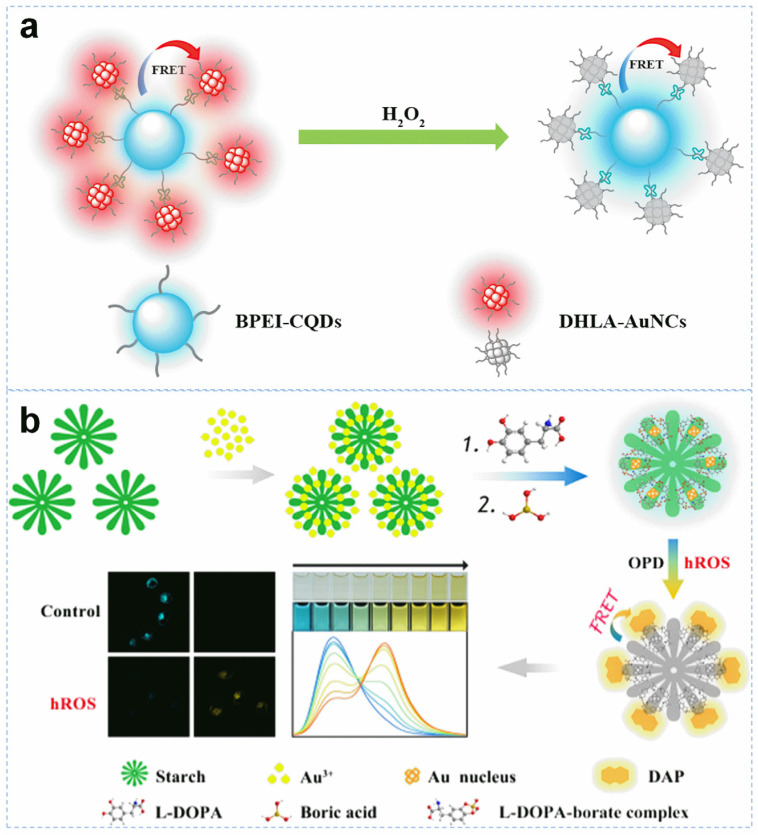
Schematic illustration of ROS/hROS detection with Au NC-based probes. (**a**) Ratiometric H_2_O_2_ detection based on a carbon quantum dot-Au NC nanosatellite. Reprinted with permission from ref. [58]. Copyright 2017, Elsevier. (**b**) Ratiometric hROS detection by integrating starch-supported Au NCs and OPD. Reprinted with permission from ref. [60]. Copyright 2020, American Chemical Society.

As mentioned above, a chemical with a high oxidizing property can quench the fluorescence of Au NCs by changing the surface state. Accordingly, reactive oxygen species (ROS), including H_2_O_2_, superoxide anion radical (·O_2_^−^), hydroxyl radical (·OH), hypochlorite radical (ClO^−^), and peroxynitrite (ONOO^−^), with a strong oxidation capacity may also induce the fluorescence quenching. Specifically, ·OH, ClO^−^, and ONOO^−^ are usually called highly ROS (hROS) due to their ultrastrong oxidation capacity. The content of ROS are generally an indicator for the oxidative stress. The detection of H_2_O_2_ with a Au NC was realized through fluorescence quenching or ratiometric change. As indicated in Li’s report, the dual-emissive nanosatellite consists of carbon quantum dots, and Au NCs allowed ratiometric H_2_O_2_ detection [58]. The red emission of dihydrolipoic acid-protected AuNCs was largely affected by the introduced H_2_O_2_, while the blue emission of carbon quantum dots showed ignorable variation (Figure 3a). The nanosatellite endowed a linear H_2_O_2_ perception in the concentration range from 5.0 to 80 nM and provided a LOD of 2.9 nM. The imaging of intracellular and endogenous H_2_O_2_ in HeLa cells was also achieved. To achieve high sensitivity toward ROS detection, the stability of Au NCs should be relatively poor. However, there was no work that indicated the correlation between a gold atom and LOD toward ROS. On the basis of the reported works, we believe the surface charge state would be a critical factor that regulates the reactivity toward ROS and decides the LOD. The surface charge state is related to the atom number-mediated geometry and surface ligand.

The discrimination of hROS is important for indicating the oxidative environment, and much research focused on hROS detection has been reported. For example, the dual-ligand-protected Au NCs could be used for sensing ·OH, ClO^−^, and ONOO^−^ [59]. In Xie’s report, the ligand decoration could promote the stability of GSH-capped Au NCs. The peptide and GSH co-modified Au NCs were against H_2_O_2_ and ·O_2_^−^ but showed a response toward ·OH, ClO^−^, and ONOO^−^. The hROS imaging was realized in living cells and even zebrafish.

In addition to the direct oxidization of Au NCs, other substances showing redox-related fluorescence variation can also be used to construct ratiometric sensing systems. In particular, *o*-phenylenediamine (OPD) can be oxidized to 2,3-diaminophenazine with a strong yellow emission, making it a good substrate for catalytic application. By integrating Au NCs and OPD, Fang et al. constructed a ratiometric system for the specific monitoring of hROS [60]. In their work, the blue emissive and starch supported Au NCs acted as the donor, while the fluorescent 2,3-diaminophenazine product behaved as the acceptor (Figure 3b). With the introduction of hROS, the yellow emission increased, accompanied by a decreased blue emission, leading to ratiometric fluorescence variation. Similarly, Quan et al. reported a ratiometric hROS imaging system with the use of cellulose nanocrystal-stabilized Au NCs and OPD [61]. The hROS-induced fluorescence variation imaging in HepG2 cells and L929 cells was achieved. Interestingly, multiple-ROS discrimination was reported through linear discriminant analysis.

Despite the development of Au NC-based sensors, the detection of ROS still poses significant challenges due to their inherent short lifetimes and reactive nature. The transient existence of ROS makes it difficult to capture and quantify them. As a result, this limitation hinders the accurate assessment of ROS levels in biological systems, which is crucial for understanding their roles in cellular signaling, homeostasis, and disease pathogenesis. Ongoing research efforts are focused on developing highly reactive and sensitive probes that can overcome these limitations and provide accurate, real-time information on ROS levels in biological systems.

### 2.4. Biomacromolecules

Biomacromolecules including peptide, protein, DNA, and RNA are critical to the biological process and can act as biomarkers for indicating various diseases. To achieve a rapid analysis of these biomacromolecules, various Au NC-based systems have been reported by diverse principles. Since Au NCs are fluorescent, they can be used as fluorescence tags. For example, Zhuang et al. reported a dot-blot immunoassay based on immunoglobulin G-functionalized Au NCs (IgG-Au NCs) [62]. The IgG-Au NCs showed a high binding affinity to goat anti-human IgG, providing a potential application for antigen/antibody sensor design with low nonspecific adsorption.

**Figure 4:**
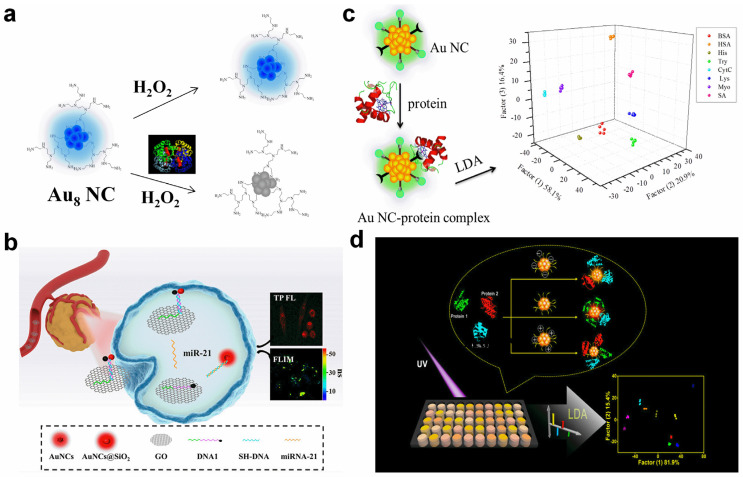
Schematic illustration of biomacromolecule detection with Au NC-based probes. (**a**) Turn-off detection of hemoglobin using hPEI-capped Au_8_ NCs in the presence of H_2_O_2_. (**b**) Fluorometric and lifetime imaging-based miRNA-21 detection with the combination of Au NCs and GO. Reprinted with permission from ref. [65]. Copyright 2023, American Chemical Society. (**c**) Discrimination of eight proteins using a Au NC-based sensor array. Reprinted with permission from ref. [66]. Copyright 2015, American Chemical Society. (**d**) Protein and serum differentiation with a Au NC-based sensor array. Reprinted with permission from ref. [67]. Copyright 2019, American Chemical Society.

For some proteins, the inside of a catalytic center can promote the production of reactive species, e.g., hROS. Therefore, the indirect detection of these proteins can be detected by fluorescence quenching of Au NCs. As referred in Lu’s report, the hPEI-capped Au_8_ NCs were stable in H_2_O_2_ media, while it became unstable with the introduction of ·OH [63]. With this property, they designed an on–off probe for the rapid detection of hemoglobin (Figure 4a). The mechanism is that the heme motif in hemoglobin can catalyze the occurrence of a Fenton reaction, which changes H_2_O_2_ into ·OH and destroys the structure of Au_8_ NCs. In addition, the absorption profile of a hemoglobin solution showed as partially overlapped with the emission spectrum of Au_8_ NCs, leading to the energy transfer from Au_8_ NCs to hemoglobin. The destruction of Au_8_ NCs, as well as the energy transfer, caused the fluorescence quenching. Due to the specific quenching principle, other proteins, small molecules, anions, and even metal ions could not induce similar fluorescence variation. The LOD toward hemoglobin was measured to be 5.0 nM, and hemoglobin evaluation with small deviations and good recoveries was performed in blood samples.

Tang et al. reported a fluorometric DNA sensing probe based on avidin-stabilized Au NCs (Av–Au NCs) [64]. In their report, the Av–Au NCs were first assembled via a biotin-modified DNA linker at both ends, and then the assembly was attached to magnetic beads through target DNA-mediated hybridization. After separation, the magnetic bead-related fluorescence was proportional to the DNA concentration, leading to linear variation within the DNA concentration from 0.2 nM to 20 µM. With the fluorometric measurement, the target DNA could be detected down to 0.043 nM. MicroRNA with a similar property to DNA can also be detected by such a tactic. In addition, the introduction of a fluorescence quencher was usually applied for the design of a turn-on sensor. For instance, with the combination of graphene oxide (GO) and DNA-functionalized Au NCs, Li et al. reported a turn-on miRNA-21 detection system [65]. GO with an electron-rich feature quenched the fluorescence of adsorbed single-strand DNA-functionalized Au NCs. After the introduction of miRNA-21, a double-strand DNA/RNA structure formed, which causes the liberation of Au NCs and fluorescence recovery (Figure 4b). In addition, the longer fluorescence lifetime of Au NCs was also found in intact MCF-7 cells, providing a two-model microRNA sensing platform.

For multiple targets’ identification, linear discriminant analysis was usually involved. Yuan et al. designed and synthesized eight dual-ligand-functionalized Au NCs with similar fluorescence characters but diverse surface properties by the same synthetic protocol [66]. The different hydrophilicities, charge states, and functional groups contributed various binding affinities toward eight proteins. Therefore, each protein caused the unique fluorescence variation patterns, with eight Au NC-comprised sensing units. Through linear discriminant analysis treatment, eight proteins with different isoelectric points and molecular structures were well classified (Figure 4c). The blind sample tests demonstrated the high accuracy of the proposed sensor array. With a similar strategy, Luo et al. presented the discrimination of proteins and serums using peptide-functionalized Au NCs [67]. They found that the bind affinity of Au NCs to proteins could be affected by solution pH. By recording the fluorescence variation at different pH values, they also constructed a sensor array toward multiple proteins. With the differentiation of serums, patients from breast cancer, rectal cancer, severe osteoarthritis, and healthy people could be completely identified from each other (Figure 4d). It should be noted that the molecular structures of biomacromolecules are more complicated than that of small molecules, and the same unit exists (amino acid or nucleotide) in different biomacromolecules. To design specific nanoprobes, it would be better to recognize the specific region (e.g., unique sequence), or 3D conformation or catalysis-related intermediates or products.

### 2.5. Cancer Cells

The early detection and accurate diagnosis of cancer cells are crucial for effective cancer treatment [68]. To realize cancer cell detection, a general route is to target the characteristic biomarkers on the membrane or inside cell. For instance, using DNA MUC1 aptamer-modified Au NCs (MUC1-AuNCs), Feng et al. reported the recognition of 4T1 cancer cells by targeting mucin overexpression [69]. It is well known that folate receptor alpha protein overexpression usually happens in cancer cells, making folic acid a good marker for a targeting application. With folic acid modification, Hada et al. developed a NIH:OVCAR-3 human ovarian adenocarcinoma cell-targeting approach [70]. Through confocal fluorescence lifetime imaging, the intracellular localization of a Au NC probe was monitored.

Similarly, with sulfated oligo-iduronic acid ligand functionalization, Chandra et al. found that the encapsulated Au NCs could penetrate the extracellular matrix and were selectively taken up by EGFR-overexpressed cancer cells [71]. Through U_11_ peptide modification, Chiechio et al. realized pancreatic tumor cell targeting [72]. The U_11_ peptide facilitated penetration into the pancreatic cell nuclei rather than cell membrane, and bright emission was observed around cell nuclei. In addition, the biodistribution of Au NCs in early embryos and free-swimming juvenile zebrafish was also investigated. Zhu et al. found that surface modification with fluorinated polymers could enhance the tumor permeation, and the weakly acidic tumor microenvironment would cause the aggregation of Au NCs and thus facilitate retention [73]. As a consequence, fluorescence imaging with long-term stability was obtained.

Different to the above tactics, Ouyang et al. proposed long-term cellular tracking by intracellularly biosynthesized Au NCs [74]. In their study, the DNA nanoribbon could promote the formation of Au NCs in tumor cells and showed a 4-fold enhancement in comparison to free Au NCs. The fluorescence imaging could be conducted even after 48 h, revealing the high stability of endogenous Au NCs. The subcellular localization of Au NCs in MCF-7 cells was demonstrated by the fluorescence colocalization technique. In addition, such an approach could be applied for the synthesis of other metal NCs. Compared to ions or molecules, cells are more complicated, and the high specificity toward the expected cancer cells is difficult. To realize the selective targeting, it would be applicable with proper surface functionalization. Beyond intracellular markers, the specific targets on a membrane can also be considered, such as proteins, phospholipids, and saccharides.

### 2.6. Bacteria

**Figure 5:**
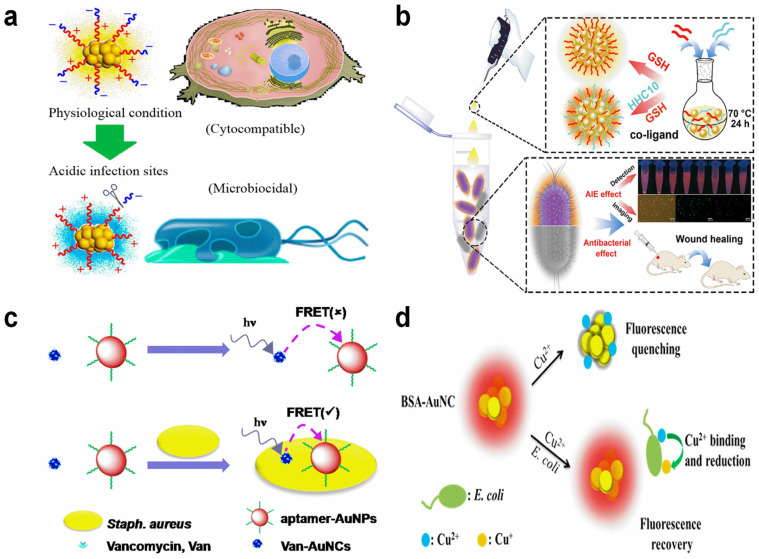
Schematic illustration of bacteria recognition with Au NC-based probes. (**a**) Imaging and killing of bacteria with antimicrobial peptide-capped Au NCs. Reprinted with permission from ref. [75]. Copyright 2019, American Chemical Society. (**b**) HHC10 antimicrobial peptide modification-mediated bacteria recognition. Reprinted with permission from ref. [77]. Copyright 2022, Elsevier. (**c**) Selective detection of *Staphylococcus aureus* with a dual-recognition strategy based on fluorescence resonance energy transfer (FRET). Reprinted with permission from ref. [78]. Copyright 2017, American Chemical Society. (**d**) On–off–on detection of *Escherichia coli* by a competition strategy. Reprinted with permission from ref. [79]. Copyright 2018, American Chemical Society.

Microbial infection-related diseases cause great attention and have become a global healthcare problem, making the rapid and sensitive detection of bacteria important. For bacteria analysis, the normal strategy is to functionalize Au NCs with recognition substrates, such as antibacterial peptide, aptamer, and negatively charged molecules. Through specific binding, the Au NCs are bonded to the surface of bacteria and provide a strong emission during imaging. Antibacterial peptides are the star markers and have been widely used for surface modification. The positive charge of antimicrobial peptides facilitates the binding between Au NCs and a negatively charged bacterial membrane. As reported in Pranantyo’s work [75], the cysteine-terminated antimicrobial peptide directly formed Au NCs could be used for bacterial labeling (Figure 5a). The antimicrobial peptide not only served as a stabilizer, but also acted as a reducing agent. The collected bacteria were represented with the fluorescence signal. That is, the higher the bacterial concentration, the higher the fluorescence. With this approach, both *Escherichia coli* and *Staphylococcus aureus* could be readily detected. Similarly, Shen et al. reported the detection and killing of *Escherichia coli* and *Staphylococcus aureus* with antimicrobial peptide (CWFWKWWRRRRR, CWR11)-stabilized Au NCs [76]. In Wang’s report [77], the modification of an antimicrobial peptide (HHC10) promoted the rapidly and selectively detected Gram-positive bacteria (Figure 5b). The HHC10 functionalization showed a high binding affinity to DNA and assisted the generation of ROS, thus leading to membrane damage and broad-spectrum antibacterial activity.

Beyond the direct imaging of bacteria, an energy transfer pathway has also been applied for effective bacteria sensing. For instance, Song et al. proposed a dual-recognition strategy with vancomycin-modified Au NCs and aptamer-functionalized gold nanoparticles [78]. In their work, the vancomycin-modified Au NCs showed a bright emission in solution. However, after the simultaneous binding to *Staphylococcus aureus*, the Au NCs got close to gold nanoparticles, leading a dramatic emission inhibition due to fluorescence resonance energy transfer (Figure 5c).

In addition to functionalization with the recognition substrates, direct sensing of bacteria with thiolate-capped Au NCs was also reported. As referred to in Yan’s work [79], the red emission of BSA-capped Au NCs was largely quenched by Cu^2+^. However, the Cu^2+^ trended to interact with *Escherichia coli* and became Cu^+^ via reduction. The Cu^2+^ binding competition induced fluorescence recovery (Figure 5d), showing an on–off–on bacteria detection event. Based on this fluorescence variation, the rapid determination of *Escherichia coli* in an artificial contaminated water sample was achieved in 30 min. The LOD was established to be 100 CFU/mL. The diverse surface charge states, as well as the different metabolic pathways, usually make selective bacteria recognition difficult. In addition to genetic DNA/RNA, metabolites could also act as symbols for bacteria. For example, the metabolic product 2,6-dipicolinic acid has been identified as a unique anthrax biomarker [80]. The perception of genetic materials or metabolites is functional for bacteria detection.

In a word, the analyte–Au NC interactions and/or target–ligand interactions would induce the variation of a dispersive state, charge transfer, and nanostructure evolution, and thus change the fluorescence properties of Au NCs. On the basis of the fluorescence mutation, the detection of diverse analytes has been successfully realized. To facilitate the reading, the detection performances of partial Au NC-based probes are summarized in Table 1.

## 3. Au NC-Based Therapeutic Applications

As one of the most important global diseases, cancer causes severe health issues and leads to growing death. It is thus appealing to develop effective therapeutic methods and materials to defend against cancer. Using Au NCs as the therapeutic agents or supports, the therapeutic applications can be majorly divided into three categories: photodynamic therapy, photothermal therapy, and as a drug carrier [81,82,83,84,85].

### 3.1. Photodynamic Therapy

Photodynamic therapy (PDT) is a typical light-based cancer therapy technique, which usually requires the participation of photosensitizers [86,87]. Generally speaking, the photosensitizers are non-toxic chemicals/materials. However, after activation with light, they produce ROS, mostly singlet oxygen (^1^O_2_) or ·O_2_^−^, which cause irreversible cell damage and reduce biological activity, then result in tumor regression.

**Figure 6:**
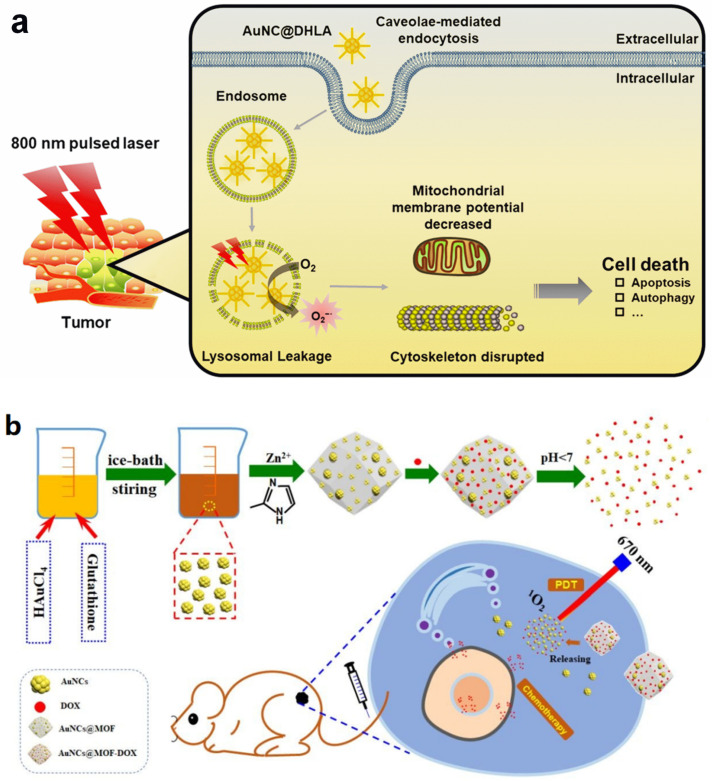
Schematic illustration of PDT applications with Au NCs. (**a**) DHLA-Au NC-mediated PDT through the production of ·O_2_^−^. Reprinted with permission from ref. [88]. Copyright 2020, American Chemical Society. (**b**) pH-activated PDT and chemotherapy by metal–organic framework-encapsulated AuNCs and doxorubicin. Reprinted with permission from ref. [90]. Copyright 2020, Royal Society of Chemistry.

Han et al. discovered that the dihydrolipoic acid-coated Au NCs (DHLA-Au NCs) possessed a large penetration depth and a high, two-photon absorption cross-section [88]. Thus, they reported an efficient in vivo two-photo PDT system based on DHLA-Au NCs. According to their study, the DHLA-Au NCs entered the tumor cell through an endosome pathway and promoted the generation of ·O_2_^−^ under light irradiation. The produced ROS decreased the mitochondrial membrane potential, disrupted the cytoskeleton, and, finally, induced cell death (Figure 6a). With a high efficiency of ·O_2_^−^ generation, DHLA-Au NCs was also used for an in vivo antitumor application. The results showed that the morphology of most tumor cells did not maintain a 800 nm fs laser light, revealing high therapeutic efficiency.

Santhakumar et al. proposed silver-doped Au NCs with high inherent photochemical activity and therapeutic efficiency through stabilization with two tripeptides [89]. The silver doping contributed strong fluorescence, while the tripeptide modification endowed the rapid generation of ^1^O_2_. The two tripeptides’ modification showed high PDT efficiencies toward MCF-7 and MDA-MB-231 cells and triggered the loss of mitochondrial membrane potential. Compared to MCF-7 cells, PDT treatment displayed higher efficiency in MDA-MB-231 cells. They performed the PDT experiments on different cancer cells.

The metal–organic framework with a rigid structure can be a solid support to assemble Au NCs and enlarge reactivity. To further enhance therapeutic efficiency, Zhang et al. developed a pH-responsive metal–organic framework-encapsulated AuNC with doxorubicin loading [90]. The structure of the metal–organic framework was pH-responsive, which collapsed within an acidic tumor microenvironment, leading to the release of AuNCs and doxorubicin (Figure 6b). In this case, both PDT and chemotherapy were activated, resulting in high therapeutic efficiency. The tumor microenvironment-mediated doxorubicin release rate could reach to 77.1%. In addition, the generation of ^1^O_2_ was realized by 670 nm of laser irradiation. The integration of PDT and chemotherapy largely promoted the therapy of tumors.

To further reduce the phototoxicity during PDT treatment, the development of NIR-II photosensitizers has attracted widespread research interest in subsequent research. For example, Dan et al. prepared BSA-capped Au NCs with multiple functions, including NIR-II emission, catalase-like activity, and PDT character [91]. With 4T1 tumor-bearing mouse models, they found that the high signal-to-background ratio could be realized. In addition, the catalase-like activity enabled an oxygen self-supplied capability, yielding boosted PDT efficiency. They also demonstrated a bacterial infection treatment with proposed BSA-capped Au NCs, indicating wide PDT applications against cancer and bacterial infections. For exploring advanced Au NC-based PDT agents, there are three factors should be considered. 1. Large NIR-II light absorption to prevent phototoxicity. 2. High yield of ^1^O_2_ generation for effective therapy. 3. Low cytotoxicity to reduce the dosage.

### 3.2. Photothermal Therapy

In some cases, the adsorbed light (i.e., NIR region) energy can also be converted into heat energy through non-irradiative process, leading to the increase of temperature around light absorbers, e.g., metal nanoparticles. Such a light-induced heating is called photothermal therapy (PTT) and has been used for killing tumor cells. Involving the use of light-absorbing properties of Au NCs to generate heat, Au NCs are also applicable for the use in PTT applications. That is, when exposed to NIR light, the Au NCs absorb the light energy and convert it into heat energy, leading to a rise in temperature in the tumor microenvironment, destroying cancer cells. In most cases, the Au NCs are designed to concentrate in the tumor region, allowing for targeted therapy. This type of localized heating causes damage to the cancer cells, leading to their death, while sparing the surrounding healthy cells. Such a treatment is minimally invasive and has fewer side effects than traditional cancer treatments.

The PTT effect of a single Au NC was reported to be low and was difficult to support in therapeutic applications. Zhao et al. found that the sodium alginate-stabilized Au NC aggregates possessed high PTT efficiency in comparison to solely Au NCs [82]. The-pH controlled aggregation was employed in tumor treatment through activated PTT. The therapeutic mechanism was summarized as follows: the Au NCs were well dispersed in a normal environment with inactive PTT; however, it trended to form aggregates after entering the acidic tumor area because of the pH-responsive sodium alginate, and the PTT effect was turned on. Interestingly, the blood circulation of Au NCs was prolonged by a sodium alginate modification, which benefits the tumor accumulation of Au NCs.

Despite the direct PTT with Au NC aggregates, the total efficiency is still low. To further enhance the PTT effect of Au NCs, the surface modification of photosensitizers was explored. Byun et al. reported that surface functionalization with methylene blue could enhance the therapeutic ability of Au NCs [92]. Similarly, with MXene nanosheet modification, Singh et al. also proposed the highly effective PTT treatment of cancer cells using folic acid-capped Au NCs [93]. The NIR absorption character of MXene nanosheets largely boosted the PTT efficiency and endowed the conjugates with a high photothermal conversion efficiency of 43.51%. The PTT-induced apoptosis contributed to in vitro cell death, and a high PTT effect was presented in various cell lines. To ensure the effectiveness and safety of PTT, the full consideration of a few factors is necessary. 1. Precise control of the intensity and time of light irradiation. 2. Selection of appropriate light sources and wavelengths. 3. Consideration of the characteristics of the target tissue.

### 3.3. Drug Carrier

In addition to the direct photo-induced generation of ROS and heat, being a drug carrier is another way to realize effective therapy. Au NCs with a small size and high surface area-to-volume ratio provide a great nanointerface to deliver organic molecules and biomacromolecules with functional groups, e.g., carboxylic and amino groups. Through physical adsorption or chemical modification, drugs can be successfully loaded. After entering the cell or approaching the specific environment, these cargos release from the Au NC surface by pH/target-weakened drug–Au NC binding. The free drugs would interact with the organelles or facilitate the generation of ROS and, thus, promote apoptosis and kill the tumor cells.

**Figure 7:**
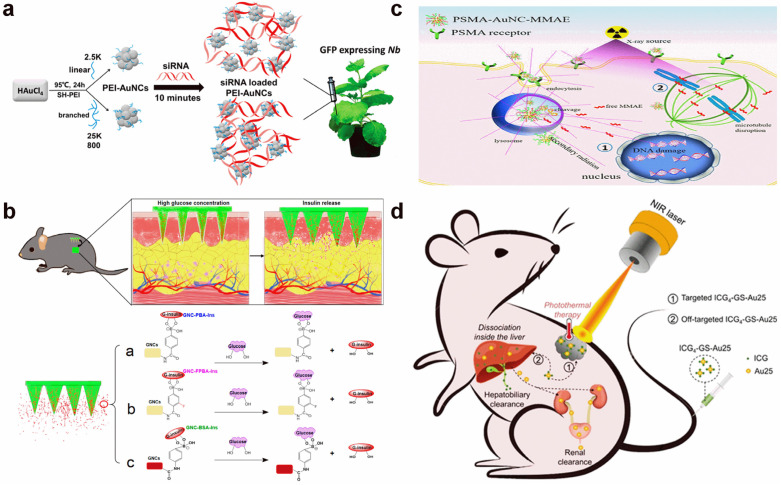
Schematic illustration of drug carrier-based therapy with Au NCs. (**a**) Gene knockdown by siRNA-loaded Au NCs. Reprinted with permission from ref. [94]. Copyright 2021, American Chemical Society. (**b**) Insulin carrier for glucose-responsive type 1 diabetes therapy. Reprinted with permission from ref. [95]. Copyright 2020, American Chemical Society. (**c**) Cleavable monomethyl auristatin E anchor for protease-activatable chemoradiotherapy of prostate cancer. Reprinted with permission from ref. [96]. Copyright 2022, American Chemical Society. (**d**) Indocyanine green adsorption-enhanced PTT. Reprinted with permission from ref. [97]. Copyright 2020, American Chemical Society.

Through the surface adsorption of siRNA, Zhang et al. reported the efficient gene knockdown of plant cells [94]. The hPEI-functionalized Au NCs with a positive charge acted as a good carrier to adsorb negative siRNA and prevent its degradation by RNase. In addition, the positive nature and amino rich character made hPEI-functionalized Au NCs easily penetrate the plant cell wall and membrane (Figure 7a). Taken together, high gene knockdown efficiencies above 75% were achieved for both the green fluorescent protein gene and ROQ1 gene.

Chemical conjugations of protein and cytotoxin onto the Au NC surface have also been reported. The insulin-conjugated Au NCs with a glucose-responsive character were prepared through borate ester chemistry [95]. They found that the closed-loop insulin release could be reached both in glucose solutions and in vivo in type 1 diabetic mice. The glucose could also bind to boric acid to form a borate ester, and such a competition reaction broke boric acid–insulin binding and led to the release of insulin (Figure 7b). By the cleavable anchor of monomethyl auristatin E, Luo et al. reported the targeted chemoradiotherapy of prostate cancer using Au NCs [96]. The anchored monomethyl auristatin E on the Au NC surface did not show strong toxicity due to the chemical modification. However, after entering the cell, the protease would cut off the cleavable linker and release the monomethyl auristatin E due to the over-expression of a prostate-specific membrane antigen (Figure 7c).

The photosensitizers with Au NC delivery also showed a high therapeutic efficiency. Jiang et al. reported that the conjugation of indocyanine green and GSH-capped Au_25_ NCs could largely enhance the photostability and tumor targeting of indocyanine green [97]. Meanwhile, its photothermal performance was improved and blood circulation was prolonged (Figure 7d). The synergic effect of these factors contributed to high PTT therapeutic efficacy. Therefore, under weak NIR light irradiation, tumor growth was completely suppressed by these conjugates.

## 4. Conclusions and Perspectives

In this review, we have summarized the very recent applications of Au NCs focused on biosensing and therapy. The biosensing assays are dependent on the fluorescence variation of Au NCs and can be classified by three types: turn-off, turn-on, and ratiometric change. With proper modification, in vitro and in vivo biosensing have been realized with high sensitivity and specificity. Au NC-involved therapy has been divided into three categories: PDT, PTT, and as a drug carrier. The therapeutic efficiency is related to both intrinsic characters of Au NCs and properties of surface cargos. With a synergistic interaction, partial systems show boosted therapeutic efficiencies in cellular conditions and organisms.

As one the most biocompatible metal fluorophores, ultrasmall Au NCs with renal clearable features are important in reducing toxicity. Despite the reported numbers of Au NC-based biosensing and therapeutic systems, the development of versatile Au NCs with unique properties may still attract great interest in future research. To take Au NCs to the next level, function-programmable Au NCs with the advantages of easy preparation, high stability, and strong emission are needed. In addition to idiographic substances, the detection/visualization of environmental variation, specific interaction, and essential process is also an important aspect [98,99]. Moreover, through proper design, synergistic interactions with boosted therapeutic performances are an interesting research direction [100,101,102,103]. The understanding of Au NCs’ potential in treating newly discovered diseases may make the world better [104,105]. Taking this into consideration, future works on Au NCs can be conducted to address the following challenges:

- Highly fluorescent Au NCs in the NIR II region.

NIR II window (1000–1700 nm) light has strong penetration in biological tissues and causes minimal damage to the organism. In addition, PDT and PTT are usually performed with NIR light sources. Therefore, Au NCs that can emit light within this range are particularly suitable for deep tissue imaging, providing powerful tools for disease diagnosis and treatment.

- Ultrastable Au NCs against ROS and hROS.

Ultrastable Au NCs against ROS and hROS can maintain their integrity and functionality under oxidative conditions, ensuring their reliability in complex biological environments. Additionally, their stability and targeting capabilities could be preserved, which endow efficient delivery and imaging.

- Super-resolution imaging of single Au NCs.

Super-resolution imaging is an advanced technique that allows for the visualization of individual Au NCs with unprecedented precision and clarity, allowing for a detailed understanding of their behavior at the nanoscale. By visualizing single Au NCs with a high resolution, it becomes possible to assess their structural integrity, size distribution, and surface properties. This may open up new possibilities for discovering their potential in various applications.

- The mechanism of the aggregation-enhanced emission (AIE) of Au NCs.

Recent studies reveal that the Au NCs capped with different thiolate ligands possess AIE characters, and GSH-stabilized Au NCs and copper nanoclusters also show AIE properties. The understanding of the origination of the AIE character of Au NCs is essential for gaining fundamental insights, enhancing photoluminescence applications, controlling and manipulating the emission properties, and potentially expanding the range of applications.

- Nano–bio interface-enhanced enzymatic analysis.

The ultrasmall size and high surface energy offer a precise interaction between Au NCs and a protein enzyme, which may create hybrid systems that exhibit enhanced enzymatic activity. This enhanced enzymatic analysis offers several advantages. Firstly, it can increase the sensitivity and selectivity of enzymatic reactions, enabling the detection of low-concentration analytes or discriminating between closely related molecules. Secondly, the use of Au NCs can stabilize enzymes and protect them from denaturation or inactivation, thereby extending their shelf life and enhancing their utility in practical applications. Moreover, the nano–bio interface created by the Au NC-enzyme hybrids provides a platform for studying enzyme kinetics and mechanisms at the nanoscale.

- Waterborne and precise Au NCs from hydrophobic ligand protection.

Au NCs synthesized with hydrophobic ligands usually display precise molecular weight and uniform size distribution, which is essential for understanding the physical fundamentals of Au NC formation. However, the hydrophobility largely limits their further utilization. Therefore, waterborne and precise Au NCs derived from hydrophobic ligand protection offer a powerful tool for nanomaterial science and nanotechnology, expanding their practical applications in sensing, imaging, and therapy.

- In situ formation of Au NCs for diagnosis and therapy.

Abnormal biological status usually accompanies the generation of transient active markers; the production of Au NCs might benefit the in situ and online study of biological process monitoring. In addition, the real-time consumption of these active markers may also present further damage, leading to effective therapy.

## Figures and Tables

**Table 1:** Summary of sensing performances of partial Au NC-based detection systems.

No.	Target	Surface Ligand	Strategy	Linear Range	LOD	Environment	Refs.
1	Hg^2+^	folic-acid	Turn-off	10–1000 nM	28 nM	In vitro	[29]
2	Cu^2+^	insulin	Turn-off	0.05–1.7 μM	7.5 × 10^−3^ μM	In vitro	[33]
3	Pb^2+^	THPC/GSH	Turn-off	5 × 10^−3^–5.0 μM	2.0 × 10^−3^ μM	In vitro	[31]
4	CN^−^	hPEI	Ratiometric	0.02–1.0 μM	1.0 × 10^−4^ μM	In vitro	[35]
5	S^2−^	MUA	Turn-on	0.5–4.0 μM	3.5 × 10^−4^ μM	In vitro	[37]
6	H_2_S	GSH CDS	Turn-on	1.10–1.55 × 10^−3^ μM	--	Mouse liver	[36]
7	Cr_2_O_7_^2−^	PAMAM	Turn-on	0–55.0 μM	1.9 μM	HeLa cell	[41]
8	H_2_O_2_	CAT	Turn-on	10–80 μM	2.5 × 10^−4^ μM	In vitro	[47]
9	DA	tyrosine	Turn-off	0.1–1000 μM	10.85 × 10^−3^ μM	In vitro	[43]
10	OPs	AChE	Turn-off	1.0 × 10^−7^–1.0 × 10^−4^ μM	3.33 × 10^−8^ μM	Yeast cell	[44]
11	GSH	Manganesedioxide	On-off-on	1–300 μM	6.8 × 10^−2^ μM	Human serum	[48]
12	DA	hPEI	Ratiometric	0–25 μM	10 × 10^−3^ μM	In vitro	[54]
13	Nitrophenols	β-CDs	Array-based sensing	1–50 μM	5 μM	In vitro	[57]
14	H_2_O_2_	CQD	Ratiometric	5.0–80 nM	2.9 nM	HeLa cell	[58]
15	·OH	R_9_	Turn-off	0.2–100 μM	0.1 μM	rat blood cells	[59]
16	ClO^−^	l-DOPA	Ratiometric	0–350 μM	0.50 μM	HeLa cell	[60]
17	ONOO^−^	CNCs	Ratiometric	0–800 μM	0.79 μM	cell of zebrafish	[61]
18	Immunoprotein	IgG	Turn-on	--	6.21 × 10^−2^ μM	In vitro	[62]
19	Hemoglobin	hPEI	Turn-on	0.010–2.0 μM	5.0 × 10^−3^ μM	In vitro	[63]
20	DNA	affinity hormone	Turn-on	0.2 × 10^−3^–20 μM	0.043 × 10^−3^ μM	In vitro	[64]
21	Proteins	GSH/MUA	Array-based sensing	--	--	In vitro	[68]
22	Proteins	CMMMMM	Turn-on	0.1–50 μg/mL	--	In vitro	[67]
23	Cancer cell	MUC1	Turn-on	--	--	4T1 cancer cells	[69]
24	Cancer cell	FA-BSA	Turn-on	--	--	ovarian CC	[70]
25	Cancer cell	sulfated oligo-iduronic acid	Turn-on	--	--	regular 2D cell	[71]
26	Tumor cell	PF	Turn-on	--	--	Mouse tumor cell	[73]
27	Escherichia coli	CWR11	Turn-on	0–712 μg/mL	178 μg/ml	In vitro	[76]
28	Bacteria	HHC10	Turn-on	2.0 × 10^6^–8 × 10^8^ cfu/mL	1.7 × 10^7^ cfu/mL	In vitro	[77]
29	Bacteria	Vancomycin	Turn-on	20–1.0 × 10^8^ cfu/mL	10 cfu/mL	In vitro	[78]

-- Not available.
